# The effect of diet composition on the diversity of active gut bacteria and on the growth of *Spodoptera exigua* (Lepidoptera: Noctuidae)

**DOI:** 10.1093/jisesa/ieae031

**Published:** 2024-03-21

**Authors:** Loretta Mugo-Kamiri, Marina Querejeta, Ben Raymond, Elisabeth A Herniou

**Affiliations:** Institut de Recherche sur la Biologie de l’Insecte, UMR 7261, CNRS - University of Tours, 37200 Tours, France; Centre for Ecology and Conservation, Penryn Campus, Faculty of Environment, Science and Economy, University of Exeter, Cornwall, TR10 9FE, UK; Institut de Recherche sur la Biologie de l’Insecte, UMR 7261, CNRS - University of Tours, 37200 Tours, France; Department of Functional Biology, University of Oviedo, Asturias, Spain; Centre for Ecology and Conservation, Penryn Campus, Faculty of Environment, Science and Economy, University of Exeter, Cornwall, TR10 9FE, UK; Institut de Recherche sur la Biologie de l’Insecte, UMR 7261, CNRS - University of Tours, 37200 Tours, France

**Keywords:** cDNA metabarcoding, microbiome, insect diet, antibiotics, Lepidoptera

## Abstract

Gut microbiota plays a functional role in nutrition among several insects. However, the situation is unclear in Lepidoptera. Field studies suggest the microbiome may not be stable and is determined by diet, while in the laboratory, Lepidoptera are routinely reared on diet containing antibiotics with unknown effects on microbial communities. Furthermore, molecular approaches for the characterization of lepidopteran microbiomes rarely describe the metabolically active gut bacteria. The aim of this study was to evaluate how diet and antibiotics affect *Spodoptera exigua* (Hübner) growth and the diversity and activity of the gut bacteria community. We assessed how alfalfa and wheat germ-based diets affected larval growth, in the presence and absence of streptomycin. Alfalfa diet improved larval growth, pupal mass, and survival, but antibiotic was only beneficial in the wheat germ diet. We observed diet-driven changes in the gut bacterial communities. In the active community, the alfalfa colony was dominated by *Enterococcus* and *Rhodococcus* whereas in the wheat germ colony, only *Enterococcus* was present. In contrast, spore-forming *Bacilli* species were very common members of the DNA community. In both cases, streptomycin had a selective effect on the relative abundance of the taxa present. Our study highlights the importance of characterizing both the diversity and activity of the gut microbiota community. DNA-derived communities may include environmental DNA, spores, or non-viable bacteria, while RNA-derived communities are more likely to give an accurate representation of the diversity of active members that are potentially directly involved in the metabolic processes of the host.

## Introduction

Diet and microbiota, both as independent and interacting factors, are major drivers of insect fitness (Douglas 2015, Ng et al. 2018). A nutritionally balanced diet is key for growth and reproduction through modulation of physiological functions, metabolism, survival, as well as shaping mating attractiveness and fecundity (Cambron et al. 2019). Gut symbionts can also play integral roles in insects’ nutritional ecology (Douglas 2009). For instance, they may aid the digestion of cellulolytic plant fibers although robust in vivo evidence is still lacking (Anand et al. 2010, Xia et al. 2017). Gut microbes can also synthesize essential nutrients to supplement the host’s poor-quality diets (Chen et al. 2022, Gilliland et al. 2023) and improve the host's growth and fitness in diverse contexts (Maes et al. 2016, Somerville et al. 2019, Luo et al. 2021).

Lepidoptera (moths and butterflies) is one of the most diverse order of insects comprising over 157,000 species (Mitter et al. 2017). This order comprises of important pollinators but also some of the most destructive crop pests, that are a major threat to the global agricultural economy (Sree and Varma 2015). Some species of caterpillars are mass-reared for various purposes such as, to produce biological pesticides (Knox et al. 2014), as experimental models to screen for insecticide efficacy (Nair et al. 2019) and other biotechnological applications, making their health and fitness a priority area for research. The evaluation of host-diet-microbiota interactions in lepidopterans remains a complex area of study due to conflicting findings. Some studies suggest that diet is a key modulator of gut bacterial communities (Colman et al. 2012, Martínez-Solís et al. 2020, Mason et al. 2020, 2021, Zheng et al.2020, Muñoz-Benavent et al. 2021). For example, the gut microbial composition of *Spodoptera exigua*, reared in laboratory conditions differed between insects feeding on artificial diets and plant-based diets made from tomato and pepper plants (Martínez-Solís et al. 2020). Similarly, a dietary shift from radishes to peas led to reduced gut bacterial diversity in a laboratory-reared colony of *Plutella xylostella* (Yang et al. 2020). Furthermore, distinct differences in bacterial diversity, composition, and relative abundance were observed in the potato tuber moth, *Phthorimaea operculella*, when fed on different tissues of the same host plant (Zheng et al. 2020). Contrasting findings have also been reported where gut communities of larvae do not resemble the microbiota of the food consumed and closely related species feeding on similar diets, exhibit different gut communities (Chen et al. 2018, Hannula et al. 2019). These disparities suggest that other factors beyond diet, such as host physiology or environmental conditions can also play a substantial role in shaping the gut communities.

The functional benefits derived from possessing a gut microbiome hinge on its composition and diversity. Studies however indicate that the microbiome of caterpillars is characterized by low diversity, instability, and scant evidence to suggest the presence of a consistent core microbiota (Montagna et al. 2016, Mazumdar et al. 2021). Consequently, the question of its functional significance remains unresolved (Hammer et al. 2017, Voirol et al. 2018, Xia et al. 2020). In mass rearing, this low diversity is further subject to the routine use of multiple antibiotics to suppress microbial contaminants in artificial diets. Commonly used antibiotics include streptomycin, rifampicin, and chlortetracycline (Hoover et al. 1997, Lin et al. 2015, Chen et al. 2021). Diets lacking in antimicrobials are prone to contamination by bacteria that may produce toxins that kill the insects or cause biochemical changes in the food that affect feed utilization (Hoover et al. 1997). Antibiotics may also disrupt the host-microbiota relationships which results in both short- and long-term fitness consequences that vary across species (Lin et al. 2015, Prosdocimi et al. 2015). The choice of antibiotic is therefore a delicate balance between good inhibition of diet contamination and minimizing toxicity to the insect. In some lepidopterans, antibiotic-related perturbation of the gut microbiota has little impact on larval growth and survival, as in *Danaus chrysippus* and *Ariadne merione* (Lepidoptera: Nymphalidae) (Phalnikar et al. 2019). In others such as the oriental fruit moth, *Grapholita molesta* (Busck) (Lepidoptera: Tortricidae), antibiotics negatively affect the larval development period and fecundity (Zhang et al. 2022). Altogether, these observations question the importance of the gut microbiome in the growth and development of lepidopterans in mass-rearing conditions.

While the microbiota’s impact on lepidopteran physiology could be significant, standardized research methods for analyzing microbial communities are not yet established. The most common approach is to use amplicon libraries of single genes (typically 16SrRNA gene in bacteria) that are derived from DNA extractions. However, the DNA-based approach does not distinguish live/active (persistent) taxa from dead/dormant (transient) members of the microbial bacterial community (Hammer et al. 2017). A limited number of studies have characterized the active gut bacterial profiles of insects, especially lepidopterans, highlighting that the area remains predominantly understudied (Brinkmann et al. 2008, Reid et al. 2011, Chen et al. 2016, 2018, Rozadilla et al. 2020).

Bearing in mind that diet and microbiota interactions could play a pivotal role in shaping insect health (Zheng et al. 2017, Chen et al. 2022, Zhang et al. 2022), our goal was to find out how these 2 factors contribute to colony performance in laboratory-reared beet armyworm *Spodoptera exigua* (Lepidoptera: Noctuidae). The first aim of our study addressed how artificial diets and antibiotic affect the growth and development of *S. exigua* caterpillars and to relate this to the nutritional content of these diets. The second aim was to explore how the bacterial diversity in the insect gut varied across treatments. Finally, we tested how DNA- and RNA-based methods affected our characterization of gut bacterial communities. For the growth bioassay, caterpillars were reared on wheat germ or alfalfa-based artificial diets in the presence and absence of streptomycin sulfate antibiotic. To investigate in-depth their gut bacterial communities, we used a 16S rRNA gene metabarcoding approach from extracted DNA and RNA (cDNA). The RNA-based data reflect the metabolically active bacteria, i.e., those with higher ribosomal content, while the results from the DNA subset represent the total gut bacteria, the majority of which may be inactive, dormant, or dead cells (Reid et al. 2011). Bacteria identified in each subset can impact the host either directly by engaging in metabolic processes or they can have an indirect effect, for example, through immune priming, in the case of inactive bacteria (Dhinaut et al. 2018).

## Materials and Methods

### Diet Formulation and Nutritional Analysis

We used diets that are modifications of the Poitout diet, which has been successfully used for maintaining lepidopteran colonies and bioassays for many years (Poitout et al. 1972, Lynch et al. 1989, Cohen 2015). Two diets were tested: (i) a wheat germ-based diet, which comprised of 35.5 g wheat germ, 21.5 g agar, 120 g maize flour, 32 g brewer’s yeast, 1.3 g ascorbic acid, 4.3 g benzoic acid, 1.7 g methyl paraben (nipagin), and 1.25 g choline chloride per liter of diet; and (ii) an alfalfa-based diet containing 35.5 g alfalfa pellets, 21.5 g agar, 120 g wheat bran, 24 g brewer’s yeast, 4.3 g ascorbic acid, 1.3 g benzoic acid, 1.7 g methyl paraben (nipagin), and 1.25 g choline chloride per liter of diet. Streptomycin sulfate antibiotic at the concentration of 125 mg/L was added to the antibiotic-treated diets. This dose was chosen as it was able to inhibit microbial contamination of the diet, without adverse toxic effects on *S. exigua* and is routinely used in insect rearing (Singh and House 1970, Singh and Bucher 1971). Diet ingredients used in this study were tested for pesticide residues (Eurofins Analytics, France) and found to contain none.

To determine if the difference in growth was due to the nutritional composition of the diet, we analyzed the proximate composition of the diets at Eurofins Analytics in Nantes (France). Standard methods adapted from the European Commission (EC) regulation 152/2009 (EEC 2009) were used to determine the moisture, crude protein, crude fat, total ash, crude fiber, insoluble fiber, soluble fiber, and total dietary fiber contents of each sample. In brief, moisture content was determined by heating 2 g each of the fresh diet samples to a constant weight in an oven maintained at 105 °C. Dried samples were used in the determination of the rest of the parameters. Crude protein (% total nitrogen × 6.25) was determined by the Kjeldahl method (Kjeldahl 1883), using 2 g of sample; crude fat was obtained by hydrolyzing 5 g of each sample with hydrochloric acid in a Soxhlet apparatus and exhaustively extracting using petroleum ether (boiling point range 40–60 °C). Ash was determined by the incineration of 10 g samples placed in a muffle furnace maintained at 550 °C for 5 h. The crude fiber was obtained by digesting 224 g of sample with H_2_SO_4_ and NaOH and incinerating the residue in a muffle furnace maintained at 550 °C for 5 h.

### Growth and Development Bioassay

*Spodoptera exigua* larvae used for this experiment were reared at a temperature of 26 °C and 60% relative humidity with a 16 h:8 h light:dark photoperiod. Development parameters measured included larval and pupal mass and survival rate. Fresh egg strips containing a total of about 400 eggs were washed in 2% sodium hypochlorite solution for 60 s and rinsed twice in sterile water to reduce egg surface contamination. Egg strips were then air-dried, and placed on each of the 4 diet treatments. After 24 h, the number of hatched neonates was counted under a binocular microscope (Leica Microsystems). Each diet treatment in the survival bioassay was started off with 65 neonates and this was recorded as day one of the experiment. Larvae were reared together up to the early third instar after which they were maintained separately to prevent cannibalism. Mortality was recorded daily until the end of the experiment to calculate the survival rate. All surviving larvae were weighed at the third instar to obtain their weight gain (mg/day). On pupation, the weight of individual pupa was recorded.

Larval and pupal mass data were tested for normality using a Shapiro–Wilk test (Shapiro and Wilk, 1965) in the *stats* package in R (R Core Team 2022). Pupal mass data passed the normality test, but larval weight data were normalized by log_10_ transformation. Data were further analyzed using 2-way ANOVA followed by a Dunn’s test (Dinno 2015) for pairwise comparison using Bonferroni correction for *P*-value adjustment in the R statistical package (R Core Team 2022). Survival analysis was carried out using the *survival* and *survminer* packages in R (Kasambara et al. 2021, Therneau 2022). To assess if there were any significant effects of diet on survival, we assumed an exponential model (constant hazard), which produced the least error deviance compared to other models tested using the Akaike information criterion (AIC).

### Gut Dissection, DNA and RNA Extraction, Library Preparation, and Sequencing

We collected 97 third-instar larvae from the diet treatments and starved them overnight (46 from alfalfa with antibiotic, 33 from the non-antibiotic diet, and 19 from the wheat germ with antibiotic). Larvae were euthanized in ice and surface sterilized in 70% ethanol for 30 s and rinsed in sterile water for 30 s. The larval guts were dissected directly into DNA/RNA shield (Zymo Research) and kept at −20 °C until nucleic acid extraction. DNA and RNA were extracted from gut tissue using the *ZymoBIOMICS MagBead DNA/RNA* kit (Zymo Research) following manufacturer’s instructions. Template-free water “blanks” were processed with the same nucleic acid extraction and PCR amplification kit reagents as negative controls to detect reagent contamination. RNA was reverse transcribed to cDNA using QuantiTect Reverse Transcription kit (Qiagen) following manufacturer’s instructions. The v3-v4 region of the DNA and cDNA 16S rRNA gene was amplified using 341F (5`-CCTACGGGNGGCWGCAG-3`) and 805R (5`GACTACHVGGGTATCTAATCC-3`) tagged primers (Herlemann et al. 2011). DNA libraries were generated using a PCR cycle of initial denaturation of 95 °C for 5 min, 25 cycles of annealing at 95°C for 40 s, 55 °C for 2 min, 72 °C for 1 min, and a final extension at 72 °C for 7 min. PCR amplification success and fragment lengths (~460 bp) were checked using a 2% agarose gel electrophoresis. PCR product purification was achieved using a centrifugation protocol adding a 10:1 mix of ice-cold 100% ethanol and 3M sodium acetate; 30 min centrifugation (4,000 rpm); adding ice-cold 70% ethanol; 30 min centrifugation (4,000 rpm); drying at 80 °C and DNA was re-suspended in 40 μl molecular-grade water. Illumina sequencing adapters (Nextera XT index kit V2) were then ligated using a 9-cycle PCR of similar conditions as described above. Ligation success was confirmed by gel electrophoresis and DNA concentrations were measured using Qubit fluorometer (Life Technologies, Carlsbad, USA). Target amplicons within the gel were purified using the GeneJet Gel Extraction kit (Life Technologies). Purified pools were merged into a final pool of 40 μl (4 nM). Pooled DNA was purified using MagSi-NGSprep Plus beads (Steinbrenner Laborsysteme GmbH, Wiesenbach, Germany) and the sequencing run was performed on an Illumina MiSeq using V2 chemistry (2 X 300 bp, 500 cycles) in the sequencing center within the Biozentrum of the Ludwig-Maximilian-University in Munich (Germany). Raw sequence data are available in the NCBI Sequence Read Archive under the BioProject ID PRJNA894453.

### 16S rRNA Gene Metabarcoding Library Filtering

The quality of both forward and reverse reads from the Illumina MiSeq output was checked using the *FastQC* software (https://www.bioinformatics.babraham.ac.uk/projects/fastqc/). Forward and reverse primers were removed using the *cutadapt* software (Martin 2011). Merging then followed using the *PEAR* software (Zhang et al. 2014) setting the Phred score at 30, followed by filtering using the *vsearch v2.8.2* software (Rognes et al. 2016). Quality filtering (*fastq_maxee* = 1) was applied to discard reads that had more than 1 nucleotide error. A dereplication step was then performed to keep only unique sequences followed by a denoising step (min number of reads = 4) and indel removal (350–500 bp) to obtain a *fasta* file containing our biological communities as amplicon single variants (ASVs). These were mapped to the filtered reads to obtain the ASV table. Taxonomic classification was done using the mothur software (Schloss 2020).

### Biodiversity Analysis

As a measure of the completeness of the sampling, we computed individual ASV richness rarefaction curves using R package *vegan* (Oksanen et al. 2013). We calculated the alpha diversity using the exponential of Shannon (*q* = 1) and the inverse of Simpson (*q* = 2) indices in the R package *hilldiv* (Alberdi and Gilbert 2019). The statistical significance of the alpha diversity differences was tested using a Kruskal–Walis test. Beta diversity (community composition) was visualized through an unconstrained principal coordinate analysis (PCoA), based on the Bray–Curtis dissimilarity matrix at the ASV level using the “ordinate” function in the *phyloseq* R package (McMurdie and Holmes 2013). We tested statistical significance of the ordinations of the cDNA and DNA subsets by computing a non-permutational multivariate analysis of variance (PERMANOVA, 999 permutations) on the Bray–Curtis dissimilarity matrix using function “adonis2” from the *vegan* R package (Oksanen et al. 2013). We calculated relative read abundance, which is the proportion of every read belonging to each ASV (Deagle et al. 2019) using a customized R script based on the package *dplyr* and *ggplot2* (Wickham 2016, Wickham and Francois 2015).

## Results

### Effect of Diet Composition and Antibiotics on Insect Development and Survival

**Fig. 1.**
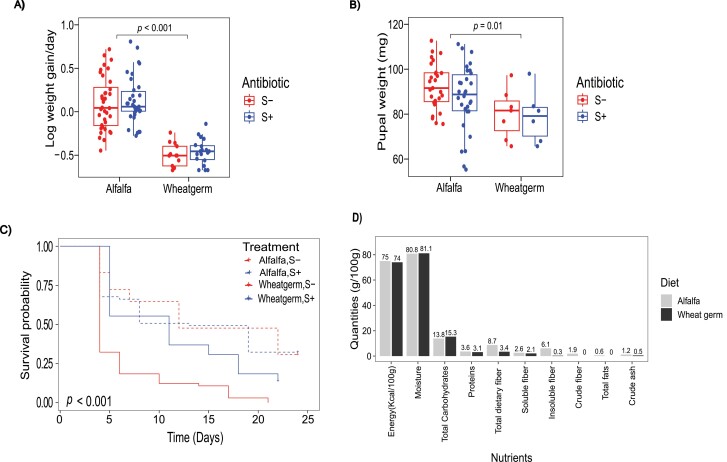
Differences in growth and survival of *S. exigua* and in diet nutritional composition. A) Larval growth and B) pupal mass in the alfalfa and wheat germ diets, with streptomycin (S+) and without streptomycin (S−). C) Probability of survival in the alfalfa and wheat germ diets with streptomycin (S+) and without streptomycin (S−). Alfalfa diet significantly improved survival compared to wheat germ (*z* = 20.58, *P* = < 2e-16). Streptomycin significantly improved survival in the wheat germ diet (*z* = 2.86, *P *= 0.0043) (dashed lines represent the alfalfa diet group, solid lines represent the wheat germ group). D) Nutritional composition of diets, alfalfa and wheat germ, showing quantities of macronutrients on dry weight basis (values on bars are in percentage).

Both larval growth and pupal mass were significantly higher on the alfalfa diet than on the wheat germ diet (*F* = 39.9; *df* = 3, 98; *P *< 0.001 and *F* = 3.13; *df* = 3, 66, *P *= 0.031, respectively) (Fig. 1A and B). In both diets, streptomycin did not have an impact on the weight (*P* > 0.1). In our survival regression model, alfalfa diet significantly improved the survival of larvae (*z* = 20.58, *P* ≤  2e-16), compared to the wheat germ diet which significantly decreased survival (*z* = −6.31, *P* = 2.7e-10) (Fig. 1C). Streptomycin alone did not show a significant effect on survival (*z* = −0.26, *P* = 0.7960), however, there was evidence of a diet–antibiotic interaction in the wheat germ diet (*z* = 2.86, *P *= 0.0043), suggesting a synergy between wheat germ diet and antibiotic in improving survival. In addition, we observed that survival in the wheat germ group without streptomycin (S−) fell below 50% by day 4, registering the highest mortality among the treatments in the early larval stages. In our analysis, alfalfa and wheat germ diets provided the larvae with similar amounts of energy, protein, and carbohydrates (Fig. 1D). There were, however, notable differences in quantities of crude fiber, insoluble fiber, and total dietary fiber, which were higher in the alfalfa diet (Fig. 1D), suggesting that fiber is the key difference between the 2 diets, and thus possibly playing a crucial role in the colony’s growth and development.

### Sequence Data Quality

A total of 16,136,196 reads were obtained from 16S rRNA gene Illumina MiSeq sequencing of DNA and RNA extracted from 97 gut samples. After denoising and indel filtering (350–500 bp), we identified 2,332 ASVs. Independent rarefaction curves tended to reach a plateau indicating that the sequencing depth was sufficient to capture the microbial diversity of the samples (Supplementary Figure S1). After filtering out samples that had less than 1,000 reads, we remained with 66 samples (25 from alfalfa with no antibiotic, 32 from alfalfa with antibiotic, and 9 from wheat germ with antibiotics) and 1,521 ASVs.

### Effect of Diet on Diversity and Relative Abundance of Gut Bacteria

**Fig. 2.**
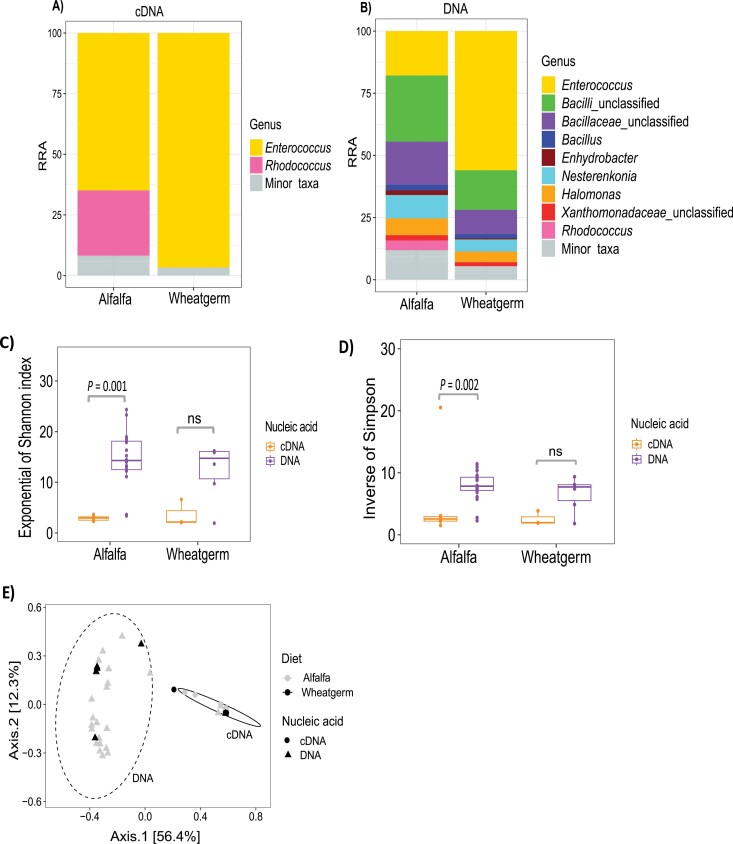
Effect of diet on *S. exigua* bacterial relative abundance and diversity. Percentage relative read abundance (RRA) in A) cDNA and B) DNA subsets in alfalfa and wheat germ diets. Alpha diversity of bacterial communities between diets as measured by C) exponential of Shannon and D) inverse of Simpson indices in cDNA and DNA subsets. E) Principal component analysis (PCoA) showing community dissimilarity between alfalfa and wheat germ diets and cDNA and DNA subsets.

In the cDNA community, the alfalfa colony was dominated by *Enterococcus* and *Rhodococcus* bacteria while the wheat germ colony was dominated by *Enterococcus* together with other low abundance taxa but notably lacking in *Rhodococcus*. In the DNA community, there were more bacteria genera present in both diets with *Bacilli* species appearing more commonly in this subset (Fig. 2A and B). Alpha diversity, as measured by Shannon and Simpson indices, did not show a significant difference in bacterial diversity between the diets (*X*^2^ = 1.4325, *P* = 0.2314). However, in the alfalfa diet, there was a significant difference in diversity between the cDNA and DNA subsets (Shannon: *X*^2^ = 10.825, *P* = 0.001, Simpson: *X*^2^ = 9.2336, *P* = 0.002) (Fig. 2C and D), indicating that the pool of metabolically active bacteria is more restricted. PCoA visualization of beta diversity showed that both communities were quite similar (no clustering by diet) although there was a clustering by nucleic acid which explained most of the variance observed between samples (Fig. 2E). Moreover, PERMANOVA (999 permutations) revealed significant differences in community compositions based on nucleic acid (*R*^2^ = 0.368, *P* = 0.001) but not on diet (*R*^2^ = 0.0242, *P* = 0.377).

### Effect of Streptomycin on Diversity and Relative Abundance of Active and Inactive Gut Bacteria

**Fig. 3.**
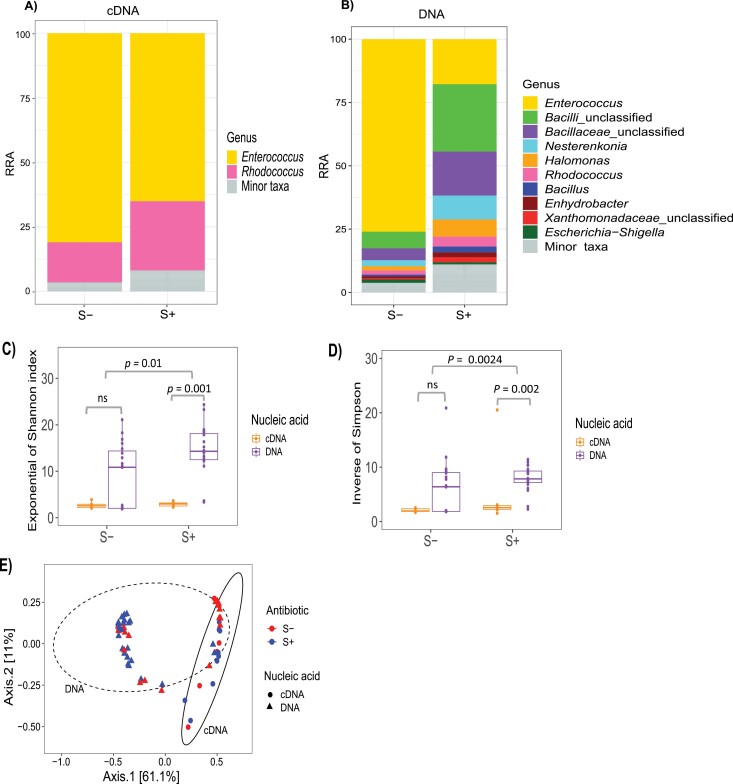
Effect of streptomycin on *S. exigua* bacterial relative abundance and diversity in alfalfa diet. Percentage relative read abundance in the A) cDNA and B) DNA subsets in streptomycin (S+) and no streptomycin (S−) treatment groups. Alpha diversity of bacterial communities between antibiotic treatments as measured by C) exponential of Shannon and D) inverse of Simpson indices in cDNA and DNA subsets. E) Principal component analysis (PCoA) showing community dissimilarity between streptomycin (S+) and no streptomycin (S−) antibiotic treatments and cDNA and DNA subsets

The effect of streptomycin on gut community structure was tested in the alfalfa diet as this treatment had higher larval survival irrespective of antibiotic consumption. In the cDNA subset, *Enterococcus* and *Rhodococcus* dominated and streptomycin only reduced the relative abundance of *Enterococcus*, with an increase in *Rhodococcus* as a result. In the DNA subset, we observed a more dramatic decrease in the relative abundance of *Enterococcus* in the presence of streptomycin (Fig. 3B). As a result, there was an apparent increase in the relative abundance of the other taxa present (Fig. 3A and B). Streptomycin treatment had a significant effect in modifying alpha diversity, as measured by Shannon and Simpson indices (Shannon: *X*^2^ = 6.704, *P* = 0.009, Simpson: *X*^2^ = 5.069, *P* = 0.024). There was a significant difference between the cDNA and DNA subset in the streptomycin treated group (S+) groups (Fig. 3C and D), while this was not the case with no streptomycin treatment (S−). The principal coordinates analysis visualization of beta diversity revealed there was no distinct clustering based on streptomycin treatment. However, there was noticeable clustering based on nucleic acid (Fig. 3E). In addition, PERMANOVA (999 permutations) revealed significant differences in community compositions based on nucleic acid (*R*^2^ = 0.229, *P* = 0.001) but not for streptomycin treatment (*R*^2^ = 0.044, *P* = 0.07).

## Discussion

Research on the growth dynamics and gut symbionts of lepidopterans is of special relevance given their status as economically important pests and the uncertainty surrounding the functions of their microbiomes (Voirol et al. 2018, Shao et al. 2023) In this study, we compared the growth and development of *S. exigua* larvae reared on alfalfa and wheat germ diets in the presence and absence of streptomycin antibiotic. Using a metabarcoding approach our study allowed the identification of the metabolically active bacteria from the pool of total gut bacteria present, and how they vary with diet and antibiotics.

Our study found that larvae fed an alfalfa diet exhibited enhanced growth, increased pupal mass, and improved survival compared to those on a wheat germ diet. This could be attributed to the higher fiber content in alfalfa. Fiber is gaining recognition as a “prebiotic” nutrient, that promotes gut microbial proliferation, which in turn may confer fitness advantages to the host organism (Hutkins et al. 2016, Savio et al. 2022). Fiber has been shown to positively influence the growth of *Tenebrio molitor* larvae, where a crude fiber content of 5% was advantageous for growth in early larval stages, whereas a 10% crude fiber level optimized overall growth and development (Li et al. 2016). A coconut fiber diet also improved larval weight, pupal weight, and flight ability of several fruit flies of the genus *Anastrepha* that are mass-reared for sterile insect technique (Aceituno-Medina et al. 2019). In addition, given that lepidopteran diets are substantially composed of plant-derived fibers, studies exploring their genomes and proteomes have revealed the presence of cellulolytic bacteria within caterpillars. While this cellulolytic activity almost certainly benefits the microbes, it is not yet known if it improves diet quality for hosts (Pauchet et al. 2008, Gandotra et al. 2018, Dar et al. 2021).

In our study, we identified *Rhodococcus*, a bacterium known for its cellulolytic activity, uniquely in the alfalfa-fed colony. A *Rhodococcus* species isolated from *Cnaphalocrocis medinalis* (Lepidoptera: Crambidae), was reported to be a strong cellulose degrader, in vitro (Biswas and Paul 2021). We, therefore, hypothesize that the elevated fiber content in alfalfa may be a key factor in favoring the growth of *Rhodococcus.* Furthermore, the main fermentation products of dietary fiber digestion are short-chain fatty acids (SCFAs) which could benefit host development (Bergman 1990). In honey bees’ gut, *Gilliamella apicola* and *Lactobacillus sp* breakdown pectin and hemicellulose in pollen to produce SCFAs such as acetate, propionate, and butyrate, which promote host growth and weight gain (Zheng et al. 2017). Our results suggest fiber content could have a considerable impact on the growth, development, and gut microbiota composition of *S. exigua* larvae. However, deciphering the mechanisms behind this observation would require further empirical research.

In the colony reared on wheat germ diet, we observed high mortality in the early larval instars (Fig. 1C). The early periods of larval development are critical to performance and fitness later in life and as such, poor diet quality during these periods may have long-term repercussions (Metcalfe and Monaghan 2001). We later observed that this treatment produced lower pupal mass (Fig. 1B) which is a proxy for fecundity (Calvo and Molina 2005), and we infer that this observation could likely be the consequence of an early life nutritional deficiency. We found that streptomycin treatment did not have any significant effect on larval and pupal mass in both diets. This contrasts with previous work in *Spodoptera litura* larvae where treatment with streptomycin increased larval growth on a different variation of a wheat germ diet (Thakur et al. 2016). Here, we observed that streptomycin promoted survival in the wheat germ diet, which could likely be due to suppression of bacteria growing specifically in this diet and which may be pathogenic to the insects. Antibiotics in diet may also play a role in suppressing mildly parasitic gut bacteria, thereby promoting insect growth and survival (Matthews et al. 2019, Mason et al. 2021). Studies have reported a high variability of the effects of antibiotics on growth and development among different, or even closely related lepidopteran species highlighting the context specificity of these interactions (Visôtto et al. 2009, Phalnikar et al. 2019, Xia et al. 2020).

In this study, *Enterococcus* was found to be the most dominant bacterial genus, particularly in the metabolically active gut community. Culturing and Sanger sequencing revealed that these isolates have high sequence similarity to *Enterococcus mundtii* (data not shown). Enterococcus is prevalent in Lepidoptera as has been reported in *Spodoptera exigua, Galleria mellonella*, *Lymantria dispar*, *Helicoverpa armigera*, and *Heliconius erato* (Broderick et al. 2004, Xiang et al. 2006, Brinkmann et al. 2008, Staudacher et al. 2016, Ramírez-Serrano et al. 2022, Upfold et al. 2023). Several *Enterococcus* species have been reported to have functional significance in insects (Mazumdar et al. 2021, Chen et al. 2022). For example, *Enterococcus mundtii* was able to increase the protection of *Tribolium castaneum* against *Bacillus thuringiensis* infection (Grau et al. 2017). In *Spodoptera littoralis*, *E. mundtii* produces mundticin which suppresses pathobionts in the gut lumen, promoting normal development of the host’s gut microbiota (Shao et al. 2017). Furthermore, *E. mundtii* was also found to be metabolically active and dominant in the eggs of *Manduca sexta* (Brinkmann et al. 2008). This is likely to benefit transmission, as metabolic activity on the egg surface may be required for effective vertical transmission of some extracellular symbionts (Voirol et al. 2018). Detecting active bacterial members can therefore also shed light on the transmission routes of gut bacteria among lepidopterans, which is an area that is not very well studied.

The second most dominant genus in the active community was found to be *Rhodococcus*, which in addition to cellulose degradation, has other diverse metabolic capabilities. These include detoxification of plant defense chemicals (Cile et al. 2001); its presence in the gut of the gypsy moth is linked to tolerance of diets with high levels of monoterpenes (Powell and Raffa 1999, Broderick et al. 2004). Alfalfa diet used in this study contained minimally processed recalcitrant plant materials which are rich in a variety of anti-herbivory phenolic compounds (Horvat et al. 2022), leading us to hypothesize that *S. exigua* larvae consuming this diet may benefit from harboring detoxifying symbionts. Furthermore, *Rhodococcus* has been implicated in other roles such as B-vitamin synthesis for diet supplementation in kissing bugs (Gilliland et al. 2023) and in pesticide degradation, where *Rhodococcus* isolates from *Helicoverpa armigera* degraded up to 43% chlorpyriphos insecticide, a potential factor in the pest’s resistance to insecticides (Madhusudan et al. 2021).

In the DNA community, which includes dormant or inactive microbes, spore-forming *Bacilli* species were found to be dominant. As spore-forming bacteria, *Bacillus sp.* are very plausible transient members of the gut community, which could partly benefit their horizontal transmission to other hosts. Moreover, some *Bacilli* such as the *Bacillus cereus* group are cadaver specialists and the gut represents a convenient niche from which to invade the insect tissues after death (Manktelow et al. 2021). The inactive gut members may have a significant role as evidenced by some probiotic studies showing that heat-killed bacteria can benefit hosts’ growth and immune response in insect rearing as well as aquaculture. Heat-killed *Pseudomonas aeruginosa* orally fed to silkworms, conferred protection against hemolymph injection of the same bacteria (Miyashita et al. 2015). A diet supplementation of heat-killed *Enterobacter sp* improved the pupal weight of irradiated *Ceratitis capitata* male flies, while heat-killed *Bacillus clausii* DE5 improved feed utilization and immune response of the grouper fish, *Epinephelus coioides* (Wang et al. 2018, Guerfali et al. 2021). A potential source of bias for the DNA community, however, is the variation in copy number of 16S genes (Pei et al. 2010, Johnson et al. 2019). Some *Bacillus* species have more than 10 copies of the 16S rRNA genes in their genome (Coenye and Vandamme 2003) which could substantially bias their relative abundance in the DNA dataset.

Streptomycin is a commonly used antibiotic in host-microbiome studies as it is considered to be relatively mild in toxicity to hosts (Lin et al. 2015). We observed a selective effect of streptomycin on our bacterial community where it decreased the relative abundance of *Enterococcus* causing an apparent increase in *Rhodococcus* (cDNA) and *Bacillus* species (DNA), a pattern also observed in other studies (Lin et al. 2015, Chen et al. 2021). *Enterococcus* in the DNA subset was particularly highly susceptible to antibiotics (Fig. 3B). If indeed these bacteria were inactive or dormant, we expect that they would be less susceptible, since dormancy is one of the strategies used by bacteria to tolerate antibiotics (Gray et al. 2019). We therefore infer that, even though these bacteria are expected to be dead or dormant, they still maintain some level of cellular activity and slow growth hence remaining sensitive to antibiotics (Gray et al. 2019).

Exposure to antibiotics can lead to significant alterations in the gut microbiota of lepidopterans and other insect orders. Such changes have been associated with adverse outcomes such as elevated mortality, reduced fecundity, prolonged development times, and reduced pupation and pupae malformation (Gaio et al. 2011, Lin et al. 2015, Raymann et al. 2017, Zhang et al. 2022). These negative effects may stem from alterations in the energy and nutrient metabolism processes that are influenced by the gut microbiome (Chen et al. 2021). For instance, in termites, antibiotic treatment was shown to cause a lasting reduction in gut bacteria diversity leading to long-term reductions in fecundity and longevity (Rosengaus et al. 2011). The use of antimicrobials not only affects host fitness but also poses a risk along the edible insect food chain, as insects are emerging as reservoirs of antimicrobial resistance (AMR) genes (Zurek and Ghosh 2014). Occurrences of AMR genes have been reported in edible mealworms and grasshoppers, which can be worsened by the use of antibiotics in rearing (Osimani, et al. 2017a, 2017b). Given the potential of resistance spreading to consumers, the careful use of antimicrobials in mass rearing is recommended (Osimani, et al. 2017a, 2017b).

Although there have been numerous studies comparing the diversity of gut communities of insects, few have described their metabolic activity. Our findings demonstrate that it would be more elucidating to use both DNA and RNA approaches for 16S rRNA gene metabarcoding studies to capture both the bacterial diversity and the taxa that have enhanced metabolic activity. Knowledge of the inactive gut community could be important for immune priming (Dhinaut et al. 2018) while the active community directly impacts metabolism and host physiology. For insects such as *S. exigua*, knowledge of the composition of both communities is important not only for mass rearing and biopesticide production but also for pest control at the farm level.

## Supplementary Material

ieae031_suppl_Supplementary_Figures_S1
