# Supplementary material for: The effect of diet composition on the diversity of active gut bacteria and on the growth of Spodoptera exigua (Lepidoptera: Noctuidae)
Source: J Insect Sci. 2024 Mar 21;24(2):13. doi: 10.1093/jisesa/ieae031 (PMC10956968; doi:10.1093/jisesa/ieae031)
Supplement: ieae031_suppl_Supplementary_Figures_S1 [file ieae031_suppl_supplementary_figures_s1.zip › Supplementary_figure1_rarefaction/Supplementary_figure1_rarefaction_caption.docx]

**Supplementary Figure S1: Sequence based rarefaction curves for *S. exigua* gut samples**. Species richness or the expected number of different species (y-axis) against number of sequences sampled (x-axis).
