# Supplementary material for: The effect of diet composition on the diversity of active gut bacteria and on the growth of Spodoptera exigua (Lepidoptera: Noctuidae)
Source: J Insect Sci. 2024 Mar 21;24(2):13. doi: 10.1093/jisesa/ieae031 (PMC10956968; doi:10.1093/jisesa/ieae031)
Supplement: ieae031_suppl_Supplementary_Figures_S1 [file Supplementary_figure1_rarefaction.pdf › Supplementary_figure1_rarefaction/Supplementary_figure1_rarefaction.pdf]

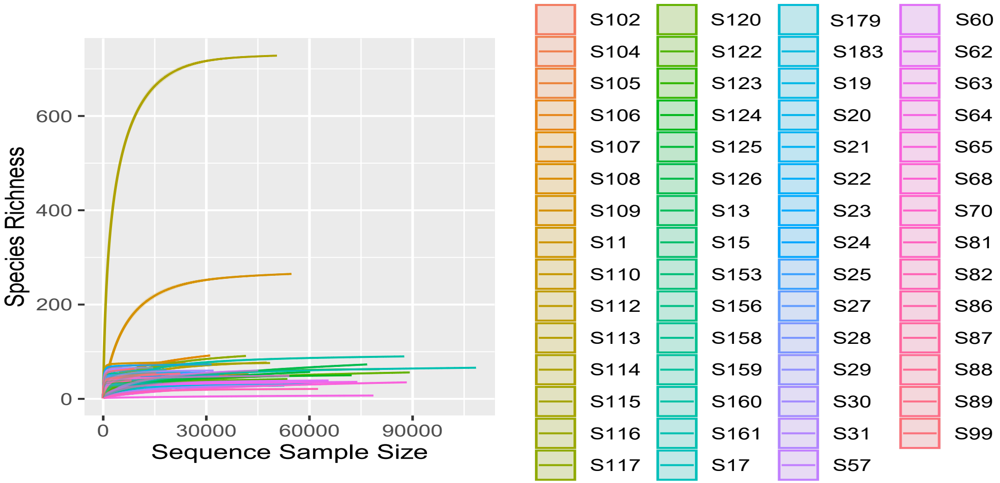
Species Richness
600
400
200
0
0
30000
60000
90000
Sequence Sample Size
S102
S104
S105
S106
S107
S108
S109
S11
S110
S112
S113
S114
S115
S116
S117
S120
S122
S123
S124
S125
S126
S13
S15
S153
S156
S158
S159
S160
S161
S17
S179
S183
S19
S20
S21
S22
S23
S24
S25
S27
S28
S29
S30
S31
S57
S60
S62
S63
S64
S65
S68
S70
S81
S82
S86
S87
S88
S89
S99
